# Resting-state abnormalities in amnestic mild cognitive impairment: a meta-analysis

**DOI:** 10.1038/tp.2016.55

**Published:** 2016-04-26

**Authors:** W K W Lau, M-K Leung, T M C Lee, A C K Law

**Affiliations:** 1 Neural Dysfunction Research Laboratory, Department of Psychiatry, The University of Hong Kong, Hong Kong; 2 Laboratory of Cognitive Affective Neuroscience, The University of Hong Kong, Hong Kong; 3 Laboratory of Neuropsychology, The University of Hong Kong, Hong Kong; 4 The State Key Laboratory of Brain and Cognitive Sciences, The University of Hong Kong, Hong Kong; 5 Institute of Clinical Neuropsychology, The University of Hong Kong, Hong Kong

## Abstract

Amnestic mild cognitive impairment (aMCI) is a prodromal stage of Alzheimer's disease (AD). As no effective drug can cure AD, early diagnosis and intervention for aMCI are urgently needed. The standard diagnostic procedure for aMCI primarily relies on subjective neuropsychological examinations that require the judgment of experienced clinicians. The development of other objective and reliable aMCI markers, such as neural markers, is therefore required. Previous neuroimaging findings revealed various abnormalities in resting-state activity in MCI patients, but the findings have been inconsistent. The current study provides an updated activation likelihood estimation meta-analysis of resting-state functional magnetic resonance imaging (fMRI) data on aMCI. The authors searched on the MEDLINE/PubMed databases for whole-brain resting-state fMRI studies on aMCI published until March 2015. We included 21 whole-brain resting-state fMRI studies that reported a total of 156 distinct foci. Significant regional resting-state differences were consistently found in aMCI patients relative to controls, including the posterior cingulate cortex, right angular gyrus, right parahippocampal gyrus, left fusiform gyrus, left supramarginal gyrus and bilateral middle temporal gyri. Our findings support that abnormalities in resting-state activities of these regions may serve as neuroimaging markers for aMCI.

## Introduction

Mild cognitive impairment (MCI) refers to an intermediate state between the cognitive changes of normal aging and the symptomatic pre-dementia stage.^1^ The prevalence of MCI is 10–20% in adults aged 65 years or above, and more than 50% of MCI patients progress to dementia within 5 years.^2, 3^ These figures draw great attention from clinicians and society at large because there is currently no effective drug that can cure dementia. MCI can be subdivided into amnestic (characterized by primarily memory impairment) and non-amnestic (characterized by executive function impairment) types based on the neuropsychological symptom profile. Amnestic MCI (aMCI) is generally regarded as a prodromal stage of Alzheimer's disease (AD), with an annual conversion rate of up to 25%.^2, 4^ This indicates the importance of early diagnosis and intervention for people with aMCI. Indeed, delayed MCI diagnosis in people with high education has been reported to impact mortality.^5^ According to the National Institute on Aging–Alzheimer's Association workgroups,^6^ the criteria for MCI include (1) a change in cognition, in comparison with the person's prior level; (2) lower performance in one or more cognitive domains that is greater than what would be expected for the patient's age and educational background; (3) independence of function in daily life is generally maintained but is less efficient compared with the past, and minimal aids may be required; and (4) the person is not demented. The standard diagnostic procedure of MCI primarily relies on subjective neuropsychological examinations that require the judgment of experienced clinicians. The development of other objectives and reliable MCI markers, such as neural markers,^2^ is therefore required.

There is a growing interest in the use of resting-state functional magnetic resonance imaging (fMRI) to explore the neurophysiological mechanisms associated with aMCI owing to its noninvasive and task-free nature. The term ‘resting state' refers to spontaneous brain activity during a passive (resting) state when one is lying quietly with the eyes closed or passively viewing a stimulus.^7^ In fact, resting-state activity is influenced by self-consciousness, ongoing thoughts, states of alertness and readiness to process stimuli from the outside world.^8^ Previous studies using fMRI have revealed a highly stereotypical pattern of spontaneous activity in a number of brain regions, namely the default mode network that manifests greater activity during passive task states compared with various active task states.^9, 10, 11^ Numerous studies have attempted to work out the difference in resting-state activity between MCI patients and healthy age-matched controls. However, the findings have been inconsistent, which might be due to the inclusion of different types of MCI patients, and/or the use of different methods across studies.

A systemic and quantitative analysis is highly warranted to delineate the meanings of the existing findings in the field. To our knowledge, only one meta-analysis has explored the difference in resting-state activity in MCI and controls.^12^ In the Supplementary Materials of this article, the authors reported reduced resting-state activity in the middle temporal gyrus, middle frontal gyrus, medial frontal gyrus and precuneus, and increased resting-state activity in a large cluster spanning the temporo-parietal parts of the brain in MCI patients from 17 independent studies. The included MCI patients were heterogeneous, including subcortical vascular MCI (svMCI) patients^13^ who might have a different pathophysiology compared with aMCI. Such heterogeneity likely impacted on the consistency of the results, given the small number of included studies. In particular, reduced resting-state activity was previously reported in the posterior cingulate cortex (PCC),^14^ hippocampal and parahippocampal regions^15^ in aMCI patients, such that the decreased PCC activity during resting state was further amplified in AD compared with aMCI.^14^ However, these findings were not confirmed by the previous meta-analysis study.^12^ Hence, the present analysis aimed to comprehensively review the abnormalities in resting-state activity in aMCI patients specifically, who have a high risk of conversion to AD, using activation likelihood estimation (ALE). An updated ALE analysis focusing on aMCI patients could provide a reasonable means to resolving discrepancies in previously reported findings.

## Materials and Methods

A comprehensive online literature search on the MEDLINE/PubMed databases was conducted, focusing on functional neuroimaging studies on MCI. Keyword searches were conducted using the following search terms: (1) ‘neuroimaging' <OR> ‘fMRI,' (2) ‘resting state' OR ‘default network' and (3) ‘mild cognitive impairment' <OR> ‘MCI.' These searches were confined to articles published in English up to March 2015, which yielded 228 original or review articles. We also searched through the reference lists of relevant review articles. From these research articles, we included studies that reported Montreal Neurological Institute (MNI) or Talairach^16^ coordinates of whole-brain contrast comparing the amnestic type of MCI and healthy controls. Two of the authors (WKWL and MKL) confirmed the inclusions of the identified studies. For included articles that reported only brain images, we attempted to obtain the MNI or Talairach coordinates from the corresponding author(s) via email (three out of eight studies replied). Studies were excluded if (1) only non-amnestic MCI or only subtypes of aMCI were included; (2) no control group was included; (3) patients had a history of neurological, psychiatric or any systemic disease that could affect cognitive functions (for example, stroke, depression, alcoholism and drug abuse); (4) *a priori* region of interest analysis or a seed-based functional connectivity analysis was conducted; or (5) the effects of medication were tested without reporting fMRI data at baseline. One study contained multiple independent patient samples; the appropriate coordinates were extracted as two separate experiments.^17^ We did not intentionally exclude studies that used a modality other than fMRI, nor focused on a particular analytic approach. As a result, the methods used in our included studies covered the following approaches: regional homogeneity, amplitude of low-frequency fluctuations and independent component analysis. Overall, 21 fMRI studies (22 experiments) reporting 156 foci were included for the ALE meta-analysis (Table 1). One of them used the arterial spin labeling perfusion MRI technique to measure resting-state abnormality in cerebral blood flow in aMCI.

GingerALE version 2.3.2 (The BrainMap Database, www.brainmap.org; San Antonio, TX, USA) was used to conduct the coordinate-based ALE analysis, which is a widely used technique for synthesizing neuroimaging data.^36, 37, 38^ ALE estimates the convergence of significant effects in terms of foci across different neuroimaging studies. Coordinates in Talairach space were imported into the software. If coordinates were reported in MNI space, the ‘icbm2tal' algorithm was used to transform them into Talairach space.^39^ For coordinates that had been transformed to Talairach space by brett-transformation, they were first ‘un-bretted' into MNI space before applying Lancaster's transformation. Imported foci were modeled as three-dimensional Gaussian spatial probability distributions using a full-width-half-maximum kernel estimated based on the corresponding experiment's sample size.^38^ These probability distributions were combined into a modeled activation map using the ‘non-additive' method.^37^ Next, the union of the modeled activation maps of each experiment was created to form the ALE image that contains the combined probability distribution of finding an activation being located at that particular voxel (that is, ALE scores). The ALE image was then thresholded using uncorrected *P*<0.001 and a cluster-level inference threshold of *P*<0.05 with 1000 permutations of simulated random data based on the characteristics of the imported data.^36^ In the cluster-level inference, contiguous voxels (that is, clusters) that exceed the cluster-forming threshold were compared against the simulated random clusters. The cluster-inference threshold approach is considered optimal because it is more stringent than uncorrected voxel-level thresholds and less conservative than conventional false discovery rate and family-wise error rate corrections. Clusters contributed by a single study only were not reported even if they exceeded the cluster-inference threshold. A total of two ALE analyses were conducted for each contrast (healthy control>aMCI or healthy control<aMCI) in all aMCI patients.

## Results

**Figure 1:**
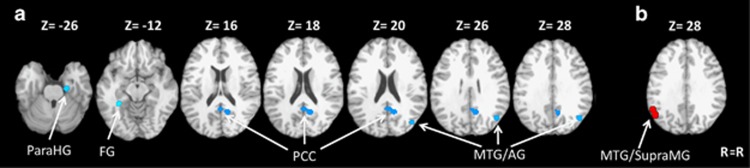
(**a**) Resting-state hypoactivation in patients with amnestic mild cognitive impairment (aMCI) compared with matched control subjects (in blue). (**b**) Resting-state hyperactivation in patients with aMCI compared with matched control subjects (in red). AG, angular gyrus; FG, fusiform gyrus; MTG, middle temporal gyrus; ParaHG, parahippocampal gyrus; PCC, posterior cingulate cortex; R, right; SupraMG, supramarginal gyrus.

aMCI patients showed decreases in resting-state activity compared with healthy controls in the right and medial PCC (Brodmann area/BA23 and 31), right angular gyrus (BA39, extending to the right middle temporal gyrus, BA19), right parahippocampal gyrus (BA35) and left fusiform gyrus (BA37) (Figure 1a and Table 2). Furthermore, aMCI patients showed increases in resting-state activity in the left middle temporal gyrus (BA39) and left supramarginal gyrus (BA40) (Figure 1b and Table 2).

## Discussion

The results of the current aggregate analyses demonstrated reduced resting-state activity in the middle temporal gyrus in aMCI compared with controls, which is consistent with a previous meta-analysis report.^12^ In addition, reduced resting-state activity was also found in the PCC, right angular gyrus, right parahippocampal gyrus and left fusiform gyrus that were not reported in the previous ALE study.^12^ Importantly, the absence of reduced resting-state activity in these regions in the previous ALE study included a heterogeneous sample of MCI patients (that is, aMCI and svMCI), suggesting that the two subtypes of MCI may possess distinct pathophysiological characteristics. In fact, increases in resting-state activity in the PCC have been reported in patients with svMCI.^13^ This further supports our hypothesis that reduced resting-state activity in the PCC may be unique to aMCI patients compared with other subtypes such as svMCI.

Episodic memory decline is the most prominent cognitive impairment marker that is used to differentiate aMCI patients from age-matched healthy controls.^40^ Brain regions including the PCC, parahippocampal gyrus, inferior and middle temporal gyri are associated with many cognitive processes, including episodic memory.^29, 41, 42, 43, 44^

Decreases in resting-state activity in the PCC were consistently observed in patients with aMCI compared with controls. According to the literature, metabolic reductions in the PCC are present in very early AD, even before a definitive clinical diagnosis,^45^ which corroborates our findings. The PCC is mainly involved in episodic memory processing.^41, 42^ Abnormal connectivity was found between the PCC and the hippocampus in early AD.^46^ Furthermore, positive correlations have been reported between the resting-state activity of the PCC and mini-mental state examination scores in MCI and AD, suggesting that resting-state activity changes in this region are associated with altered cognitive performance.^17, 23^

Decreased gray matter volume (GMV) in the PCC has been reported in both MCI and AD compared with controls using MRI volumetric measurements,^47, 48^ which could lead to a reduction in resting-state activity. To confirm that the reduction in resting-state activity of the PCC was not driven by brain atrophy, a small subgroup analysis was performed using 7 studies (38 foci) that reported resting-state abnormalities after corrected for GMV or the reported resting-state abnormalities did not overlap with the reported GMV reduction (Supplementary eTable 1). The resting-state hypoactivation in the medial PCC was maintained in aMCI patients in this subgroup analysis (Supplementary eTable 2). This finding suggests that the reduction in resting-state activity of the PCC may be independent of the GMV changes in aMCI patients. Whether the atrophy of the PCC in MCI or AD patients is driven by an early change in brain functional activity requires further study.

Reduced resting-state activity in fusiform gyrus and parahippocampal gyrus may also contribute to memory deficits in aMCI patients. For instance, the fusiform gyrus connects with the medial temporal lobe, including the parahippocampal gyrus,^49^ which has an accessory role in memory processes in healthy elderly people.^21, 50^ In addition, the previously reported positive association between the resting state of the parahippocampal gyrus and mini-mental state examination scores^15^ suggests a functional role of the resting-state activity of the parahippocampal gyrus in episodic memory.^51^

On the other hand, lower resting-state activity in the angular gyrus in the inferior parietal lobule of aMCI patients may be related to their poorer verbal working memory performance that involves short-term storage and retrieval of phonological representations.^52, 53^ Taken together, hypoactivation of the abovementioned regions is a consistent physiological change in aMCI patients, which may serve as a potential neuroimaging biomarker for aMCI.

Decreases in resting-state activity of the medial frontal gyrus and left middle frontal gyrus have been reported in a previous ALE study that included different types of MCI patients.^12^ Indeed, a previous study that reported a similar resting-state hypoactivity in the medial frontal gyrus was conducted in svMCI patients compared with controls,^13^ and the study was also included in the previous ALE meta-analysis. We did not, however, observe any significant hypoactivity in these regions in aMCI. One possible explanation for the discrepancy is that the hypoactivity of these prefrontal regions is not a defining feature of aMCI. Nevertheless, more primary studies are needed to generate a clearer picture.

Increases in resting-state activity were found in the left middle temporal gyrus and left supramarginal gyrus in aMCI patients compared with controls, which is consistent with the previous ALE study of MCI.^12^ One interpretation is that these regions are activated to compensate for the reduction in function of other brain regions. However, whether such hyperactivation of the brain is truly a compensatory response or indicates other pathological changes, such as excitotoxicity that triggers neuronal cell death, requires more empirical studies to confirm.

Five studies that compared the spontaneous brain activity between aMCI and controls were not included in the current meta-analysis due to the unavailability of the resultant coordinates. Consistent with our findings, hypoactivity in the PCC and/or precuneus was commonly reported in aMCI patients compared with controls in four out of the five studies.^54, 55, 56, 57^ In contrast to our findings, one study reported increased resting-state activity in the orbitofrontal gyrus, anterior cingulate cortex, parahippocampal gyrus, hippocampus and fusiform gyrus in multiple-domain aMCI patients, which was interpreted as a compensatory mechanism for the recruitment of cognitive resources in the patients.^55^ It is possible that multiple impairments (in memory and at least one other cognitive domain) in multiple-domain aMCI patients may trigger such compensatory responses for cognitive resources that were not present in our included aMCI patients. Another study reported increased resting-state activity of the left inferior parietal lobule in aMCI patients compared with controls, which was also interpreted as a compensatory recruitment in aMCI patients.^58^ A compensatory response may be sensitive to the stage of an illness, and in fact, the inferior parietal lobule abnormality was regarded as a sensitive marker for the transition from MCI to early stages of AD.^58^ To further understand this difference between their result and our meta-analytic findings, more information relating to the disease severity is needed.

There are several limitations in the current meta-analysis. First, although we tried to include studies that used similar diagnostic criteria for aMCI,^4, 59^ subject heterogeneity, such as subtypes of aMCI that could influence neuroimaging results,^60^ cannot be ruled out. Second, the significance level of the contributing results was not taken into consideration by the current ALE technique. Nonetheless, our study did adjust for the effect of different sample sizes. Third, different analytic approaches and imaging modalities could reveal different aspects of resting-state abnormalities in the brain.^61^ For instance, regional homogeneity and amplitude of low-frequency fluctuations measure the local synchrony and power spectrum of low-frequency signals, respectively, whereas independent component analysis separates linearly mixed low-frequency signals, which are all indirect measures of neural activity. In addition, arterial spin labeling perfusion MRI can indirectly reflect the regional brain metabolism and neural activity during resting state by measuring regional cerebral blood flow during task-free condition. Nonetheless, different analytic approaches and modalities could be complementary to each other and provide a more complete picture of the field.^61^ Given that the number of studies using each individual method is not sufficient for conducting independent ALE studies, our results represent a common pattern of resting-state abnormalities that could be revealed across imaging methodologies and have high generalizability toward understanding the neuropathology of aMCI. As shown by our findings, different analytic approaches or modalities could generate similar results, such that reduced resting-state activity was found in PCC in most of our included studies regardless of their analytic approaches or imaging modality. Finally, other confounds such as mini-mental state examination scores, age, gender and education levels that could influence resting-state activity in aMCI could not be controlled for in the current ALE analysis due to the limitation of the current analysis technique. Future studies that incorporate these variables as covariates would provide more conclusive findings on resting-state abnormality in aMCI.

## Conclusions

The current meta-analysis supports that abnormalities in resting-state activity in the PCC, right angular gyrus (extending to middle temporal gyrus), right parahippocampal gyrus, left fusiform gyrus, left middle temporal gyrus and left supramarginal gyrus are commonly found in aMCI patients. These regional abnormalities in resting-state activity may serve as neuroimaging markers for the early detection of aMCI.

## Figures and Tables

**Table 1:** List of included studies on resting state in aMCI

*Study*	*Method of analysis*	N	*Age, mean (s.d.), years*	*Education, mean (s.d.), years*	*MMSE, mean (s.d.)*	*Contrasts*	*Foci*
Xu *et al.*^18^	ASL	10 MCI	77.0 (4.5)	13.7 (2.7)	27.8 (1.5)	HC>MCI	1
		12 HC	70.0 (3.9)	15.6 (2.1)	29.6 (0.8)		
							
Sorg *et al.*^19^	ICA	24 MCI	69.3 (8.1)		27.7 (1.1)	HC>MCI	21
		16 HC	68.1 (3.8)		29.6 (0.5)		
							
Bai *et al.*^20^	ReHo	20 MCI	71.3 (3.8)	14.0 (3.1)	27.2 (1.6)	HC>MCI	9
		20 HC	69.4 (3.8)	13.8 (4.0)	28.3 (1.4)	MCI>HC	4
							
Qi *et al.*^21^	ICA	14 MCI	71.8 (7.3)	11.6 (1.0)	26.6 (0.3)	HC>MCI	7
		14 HC	70.4 (5.8)	10.0 (0.9)	28.5 (0.2)	MCI>HC	5
							
Zhang *et al.*^22^	ReHo	48 MCI	72.0 (4.4)	15.5^a^	27.0^a^	HC>MCI	9
		36 HC	71.6 (3.7)	16.0^a^	29.0^a^	MCI>HC	3
							
Wang *et al.*^23^	ALFF	16 MCI	69.4 (7.0)	10.9 (3.4)	26.5 (1.0)	HC>MCI	5
		22 HC	66.6 (7.7)	10.0 (3.9)	28.6 (0.6)	MCI>HC	4
							
Bai *et al.*^24^	ICA	26 MCI	71.4 (4.3)	13.8 (2.8)	27.2 (1.5)		
		18 HC	70.3 (4.7)	15.1 (3.1)	28.3 (1.3)	MCI>HC	2
							
Xi *et al.*^15^	ALFF	18 MCI	67.3 (7.9)	12.4 (3.2)	24.8 (3.8)	HC>MCI	3
		20 HC	64.7 (5.6)	12.2 (2.5)	28.2 (1.8)	MCI>HC	2
							
Han *et al.*^25^	ALFF	17 MCI	69.7 (7.6)	8.8 (4.0)	25.2 (3.5)	HC>MCI	6
		18 HC	66.5 (6.2)	8.4 (5.6)	29.2 (0.7)	MCI>HC	6
							
Bai *et al.*^26^	ALFF	43 MCI	72.0 (4.8)	13.6 (3.0)	27.1 (1.5)	HC>MCI	1
		30 HC	73.0 (3.5)	14.9 (2.7)	28.2 (1.4)	MCI>HC	1
							
Wang *et al.*^27^	BOLD	8 MCI	66.4 (11.0)	10.6 (3.5)	25.4 (1.3)	HC>MCI	2
		14 HC	66.1 (5.8)	11.0 (4.5)	28.0 (1.4)	MCI>HC	9
							
Zhuang *et al.*^28^	ALFF	47 MCI	72.0 (4.8)	15.9 (11.4)	27.0 (1.5)	HC>MCI	1
		33 HC	72.8 (3.4)	14.7 (2.9)	28.2 (1.3)	MCI>HC	1
							
Song *et al.*^14^	ICA	18 MCI	70.2 (7.9)	9.4 (4.8)	21.9 (5.0)	HC>MCI	1
		21 HC	65.0 (8.1)	11.0 (4.4)	28.5 (1.4)		
							
Xi *et al.*^29^	ALFF	18 MCI	67.4 (7.7)	12.3 (3.2)	25.2 (3.4)	HC>MCI	3
		18 HC	65.4 (5.8)	12.3 (2.4)	28.1 (1.8)	MCI>HC	2
							
Zhou *et al.*^30^	ALFF	17 MCI	67.0 (7.9)	11.1 (3.3)	26.4 (2.1)	HC>MCI	3
		17 HC	63.8 (5.8)	11.7 (3.0)	28.6 (1.1)	MCI>HC	4
							
Zhao *et al.*^2^	ALFF	20 MCI	65.1 (9.9)	11.8 (3.3)	25.2 (2.2)	HC>MCI	7
		18 HC	66.8 (7.4)	12.0 (2.9)	29.3 (1.2)	MCI>HC	3
							
Liang *et al.*^17^	BOLD	24 MCI	72.8 (6.6)		28.1 (1.5)	HC>MCI	2
		35 HC	74.3 (5.9)		28.9 (1.6)		
							
Liang *et al.*^17^	BOLD	29 MCI	73.2 (7.3)		27.1 (2.3)	HC>MCI	7
		35 HC	74.3 (5.9)		28.9 (1.6)		
							
Liu *et al.*^31^	ALFF	46 MCI	71.9 (4.9)	13.8 (2.6)^a^	27.1 (1.4)^a^	HC>MCI	1
		32 HC	72.8 (3.5)	14.2 (2.5)^a^	28.3 (1.1)^a^	MCI>HC	1
							
Liu *et al.*^32^	ReHo	12 MCI	59.3 (3.3)	10.5 (1.8)	26.4 (0.9)	HC>MCI	4
		12 HC	60.6 (5.8)	10.6 (2.1)	29.8 (0.4)	MCI>HC	4
							
Wang *et al.*^33^	ReHo	30 MCI	69.1 (5.8)		26.2 (2.2)	HC>MCI	5
		32 HC	70.1 (5.5)		28.1 (1.5)	MCI>HC	6
							
Zhou *et al.*^34^	fALFF	17 MCI	76.7 (5.5)		26.1 (0.7)	HC>MCI	1
		14 HC	76.3 (8.3)		29.2 (0.4)		

Abbreviations: ALFF, amplitude of low-frequency fluctuations; aMCI, amnestic mild cognitive impairment; ASL, arterial spin labeling; BOLD, blood oxygen level dependent; fALFF, fractional AlFF; HC, healthy control; ICA, independent component analysis; MCI, mild cognitive impairment; MMSE, mini-mental state examination; ReHo, regional homogeneity.

a Mean and s.d. were estimated from median or calculated according to the formulas published by Hozo *et al.*^35^

**Table 2:** Resting-state abnormalities in aMCI patients compared with controls in 21 studies

	*Side*	*Brain region*	*BA*	*Coordinates (Talairach)*			*Volume (mm*^3^)	*Extrema value*
				x	y	z		
HC>MCI	Right		23	12	−56	18		0.0150
	Medial	Posterior cingulate	23	0	−52	16	1472	0.0135
	Right		31	4	−52	24		0.0118
		Angular gyrus	39	46	−66	28		0.0189
	Right	Middle temporal gyrus	19	42	−74	22	664	0.0139
	Right	Parahippocampal gyrus	35	22	−20	−26	312	0.0190
	Left	Fusiform gyrus	37	−30	−44	−12	304	0.0155
								
MCI>HC	Left	Middle temporal gyrus	39	−50	−64	28	1280	0.0260
		Supramarginal gyrus	40	−54	−54	28		0.0192

Abbreviations: aMCI, amnestic mild cognitive impairment; BA, Brodmann area; HC, healthy control; MCI, mild cognitive impairment.
