# Supplementary material for: Resting-state abnormalities in amnestic mild cognitive impairment: a meta-analysis
Source: Transl Psychiatry. 2016 Apr 26;6(4):e790–. doi: 10.1038/tp.2016.55 (PMC4872413; doi:10.1038/tp.2016.55)
Supplement: Supplementary Information [file tp201655x1.docx]

**Supplementary Materials**

**eTable 1.** List of studies with either gray matter volume (GMV) correction or GMV reduction did not overlap with the reported brain regions

| Study | Method of analysis | N | Age, mean (SD), years | Education, mean (SD), years | MMSE, mean (SD) | Contrasts | Foci |
| --- | --- | --- | --- | --- | --- | --- | --- |
| Sorg et al. (2007)^48^ | ICA | 24 MCI | 69.3 (8.1) |  | 27.7 (1.1) | HC>MCI | 21 |
|  |  | 16 HC | 68.1 (3.8) |  | 29.6 (0.5) |  |  |
| Wang et al. (2011)^30^ | ALFF | 16 MCI | 69.4 (7.0) | 10.9 (3.4) | 26.5 (1.0) | HC>MCI | 5 |
|  |  | 22 HC | 66.6 (7.7) | 10.0 (3.9) | 28.6 (0.6) | MCI>HC | 4 |
| Bai et al. (2011)^51^ | ICA | 26 MCI | 71.4 (4.3) | 13.8 (2.8) | 27.2 (1.5) |  |  |
|  |  | 18 HC | 70.3 (4.7) | 15.1 (3.1) | 28.3 (1.3) | MCI>HC | 2 |
| Bai et al. (2012)^53^ | ALFF | 43 MCI | 72.0 (4.8) | 13.6 (3.0) | 27.1 (1.5) | HC>MCI | 1 |
|  |  | 30 HC | 73.0 (3.5) | 14.9 (2.7) | 28.2 (1.4) | MCI>HC | 1 |
| Zhuang et al. (2012)^55^ | ALFF | 47 MCI | 72.0 (4.8) | 15.9 (11.4) | 27.0 (1.5) | HC>MCI | 1 |
|  |  | 33 HC | 72.8 (3.4) | 14.7 (2.9) | 28.2 (1.3) | MCI>HC | 1 |
| Liang et al. (2014)^17^ | BOLD | 24 MCI | 72.8 (6.6) |  | 28.1 (1.5) | HC>MCI | 2 |
|  |  | 35 HC | 74.3 (5.9) |  | 28.9 (1.6) |  |  |
| Liang et al. (2014)^17^ | BOLD | 29 MCI | 73.2 (7.3) |  | 27.1 (2.3) | HC>MCI | 7 |
|  |  | 35 HC | 74.3 (5.9) |  | 28.9 (1.6) |  |  |
| Liu et al. (2014a)^57^ | ALFF | 46 MCI | 71.9 (4.9) | ^a^13.8 (2.6) | ^a^27.1 (1.4) | HC>MCI | 1 |
|  |  | 32 HC | 72.8 (3.5) | ^a^14.2 (2.5) | ^a^28.3 (1.1) | MCI>HC | 1 |

^a^Mean and SD were estimated from median or calculated according to the formulas published by Hozo and colleagues^61^.

Abbreviations: ALFF, Amplitude of Low Frequency Fluctuations; BOLD, Blood Oxygen Level Dependent; HC, Healthy Control; ICA, Independent Component Analysis; MCI, Mild Cognitive Impairment; SD, Standard deviation.

**eTable 2.** Resting-state abnormalities adjusted for gray matter volume (GMV)

|  | Side | Brain region | BA | Coordinates (Talairach) | | | Volume (mm^3^) | Extrema Value |
| --- | --- | --- | --- | --- | --- | --- | --- | --- |
|  |  |  |  | x | y | z |  |  |
| HC>MCI | Right | Cingulate Gyrus | 24 | 4 | 2 | 38 | 520 | 0.0170 |
|  |  | Superior Frontal Gyrus | 6 | 2 | 6 | 48 |  | 0.0097 |
|  | Medial | Posterior Cingulate | 23 | 0 | -52 | 16 | 368 | 0.0132 |
|  | Left |  | 31 | -6 | -54 | 20 |  | 0.0100 |

Abbreviations: BA, Brodmann Area; HC, Healthy Control; MCI, Mild Cognitive Impairment.
